# Feasibility and Acceptability of Wearable Sleep Electroencephalogram Device Use in Adolescents: Observational Study

**DOI:** 10.2196/20590

**Published:** 2020-10-01

**Authors:** Jessica R Lunsford-Avery, Casey Keller, Scott H Kollins, Andrew D Krystal, Leah Jackson, Matthew M Engelhard

**Affiliations:** 1 Department of Psychiatry and Behavioral Sciences Duke University School of Medicine Durham, NC United States; 2 Departments of Psychiatry and Neurology University of California San Francisco School of Medicine San Francisco, CA United States

**Keywords:** sleep, wearable, mHealth, adolescents, EEG, feasibility, acceptability, tolerability, actigraphy

## Abstract

**Background:**

Adolescence is an important life stage for the development of healthy behaviors, which have a long-lasting impact on health across the lifespan. Sleep undergoes significant changes during adolescence and is linked to physical and psychiatric health; however, sleep is rarely assessed in routine health care settings. Wearable sleep electroencephalogram (EEG) devices may represent user-friendly methods for assessing sleep among adolescents, but no studies to date have examined the feasibility and acceptability of sleep EEG wearables in this age group.

**Objective:**

The goal of the research was to investigate the feasibility and acceptability of sleep EEG wearable devices among adolescents aged 11 to 17 years.

**Methods:**

A total of 104 adolescents aged 11 to 17 years participated in 7 days of at-home sleep recording using a self-administered wearable sleep EEG device (Zmachine Insight+, General Sleep Corporation) as well as a wristworn actigraph. Feasibility was assessed as the number of full nights of successful recording completed by adolescents, and acceptability was measured by the wearable acceptability survey for sleep. Feasibility and acceptability were assessed separately for the sleep EEG device and wristworn actigraph.

**Results:**

A total of 94.2% (98/104) of adolescents successfully recorded at least 1 night of data using the sleep EEG device (mean number of nights 5.42; SD 1.71; median 6, mode 7). A total of 81.6% (84/103) rated the comfort of the device as falling in the comfortable to mildly uncomfortable range while awake. A total of 40.8% (42/103) reported typical sleep while using the device, while 39.8% (41/103) indicated minimal to mild device-related sleep disturbances. A minority (32/104, 30.8%) indicated changes in their sleep position due to device use, and very few (11/103, 10.7%) expressed dissatisfaction with their experience with the device. A similar pattern was observed for the wristworn actigraph device.

**Conclusions:**

Wearable sleep EEG appears to represent a feasible, acceptable method for sleep assessment among adolescents and may have utility for assessing and treating sleep disturbances at a population level. Future studies with adolescents should evaluate strategies for further improving usability of such devices, assess relationships between sleep EEG–derived metrics and health outcomes, and investigate methods for incorporating data from these devices into emerging digital interventions and applications.

**Trial Registration:**

ClinicalTrials.gov NCT03843762; https://clinicaltrials.gov/ct2/show/NCT03843762

## Introduction

Adolescence is a critical period in human development, associated with rapid physical growth, brain and cognitive development, and marked changes in social roles as youth transition from dependent roles within their family of origin to the independence of adulthood. Given the importance of these developmental processes for shaping adult behavior, interventions supporting healthy behaviors during adolescence may be particularly likely to improve and support health outcomes across the lifespan [1]. Sleep is one health behavior that undergoes significant alternations in adolescence, including shifts toward shortened sleep periods, delayed circadian rhythms, and changes in sleep architecture, including reductions in deep sleep (ie, slow wave sleep [SWS]) [2]. Poor sleep during this period is associated with risk for detrimental physical and mental health outcomes such as higher rates of obesity and onset of psychiatric disorders [3], which in turn may place individuals at risk for a range of diseases over the long-term, including cardiometabolic illness and cancer, and result in high rates of health care use and socioeconomic costs [4].

Despite the centrality of sleep to health in adolescence and beyond, sleep health among adolescents is rarely adequately assessed or treated in routine health care settings. For example, in pediatric primary care, the most common setting in which adolescents interact with health care providers, screening for sleep problems is infrequent, and when it does occur, it is typically limited in scope and subject to reporter bias (ie, one question to the adolescent or parent querying whether sleep concerns are present) [5]. Barriers to thorough and accurate sleep assessment are myriad and include lack of awareness of the importance of sleep among both patients and physicians [6] as well as the cost, invasiveness, and inaccessibility associated with polysomnography (PSG), the gold standard evaluation of sleep [7]. Thus, identifying and combating sleep disturbances in adolescence—along with their varied and profound consequences—requires a new approach that supports accurate, convenient, low-cost sleep assessment that can be deployed at the population level in real-world, health care settings.

With the rise of mobile health (mHealth), wearable technologies have offered unique opportunities to assess and shape health behaviors among adolescents [8-10]. For this age group specifically, researchers have long touted the importance of feasible, at-home collection of sleep information using devices that are acceptable to adolescents, in part due to PSG-related alterations to sleep (eg, diminished sleep quality) that occur for youth in clinic or laboratory settings [11]. This led to the widespread research use of wristworn activity monitors, such as actigraphs, to assess sleep behaviors in pediatric populations [12,13], followed by popular use of commercial grade devices, such as Fitbit or Garmin watches, that estimate sleep based on activity patterns [14]. However, because these devices estimate sleep based on other metrics (eg, movement, heart rate) rather than providing a direct measure of sleep (ie, electroencephalogram [EEG]), wristworn devices tend to underestimate some important sleep parameters, particularly in those with disturbed sleep (eg, frequency/duration of night awakenings) [13], and significantly deviate from EEG devices in their estimation of transitions between sleep stages (ie, between light, deep, and rapid eye movement [REM] sleep). Specifically, a recent study found that wristworn devices overestimate the probability of remaining in a specific sleep stage and underestimate the probability of transitioning between sleep stages [15].

To address this limitation, ambulatory (at-home) full PSG was developed and has been used in pediatric research settings [11] to characterize maturation of sleep architecture across adolescence [16,17] as well as links between sleep and healthy behaviors (eg, exercise, nutrition) [18-20], psychiatric [21] and physical [22] health, and academic and social functioning [18,23] during this developmental period. However, these devices remain expensive and require medical professionals or trained research staff to administer, restricting their utility in routine care settings and at the population health level. Consumer-grade wearables incorporating a single or a few channels of EEG have been developed to assess sleep architecture in the home environment and are relatively low cost compared with traditional and ambulatory PSG [24]. Obtaining recordings with these devices can be achieved by the subjects themselves without the involvement of a PSG technologist. This is possible because they obtain EEG data exclusively from outside the hairline (typically from the forehead and sometimes the mastoids), whereas obtaining scalp EEG data from the usual locations employed for in-laboratory studies involves placing electrodes within the hairline, which requires a PSG technologist involvement in order to obtain adequate recordings. Some provide automatic scoring of sleep behaviors and architecture that are user-friendly for patients and providers [25,26]. Among adults, consumer-grade sleep EEG wearables have been used in research and clinic settings [27,28]. However, the feasibility and acceptability of collecting sleep data using self-administered, consumer-grade sleep EEG wearables among adolescents is currently unknown.

This study had two aims. First, we examined the feasibility of collecting sleep data using a self-applied, at-home, wearable sleep EEG recording device among adolescents aged 11 to 17 years. We hypothesized that most adolescents would be capable of recording sleep EEG at home (ie, successfully recording at least 1 night), and indeed, record sufficient data to provide a stable measure of sleep architecture and physiology (ie, at least 3 nights of successful recording [29]). We simultaneously collected information about sleep behavior using a device previously associated with high feasibility and acceptability in this age group (ie, wristworn actigraph [13]) and hypothesized that most adolescents would be able to record sufficient data to provide a stable measure of sleep behavior with this method (ie, at least 5 nights of successful recording [30]). We included both sleep EEG and actigraphy measures to demonstrate the feasibility of collecting data about multiple aspects of sleep (ie, sleep physiology and architecture via sleep EEG and 24-hour sleep behavior and circadian activity patterns via the actigraphy) via wearables in this age group. In addition, multiple nights of sleep data were collected due to prior research suggesting that the stability of sleep measures increases with more nights of collection [29,30]. Second, we examined the acceptability of the wearable sleep EEG device among adolescents and hypothesized that these devices would be associated with high ratings of acceptability including a high frequency of endorsements of study satisfaction (>50%, or majority, of the sample) and a low frequency of endorsements of significant (ie, moderate to severe) device-related sleep disturbances and discomfort (<50%, or minority, of the sample). Again, we conducted a parallel query about the acceptability of a commonly used wearable in this age group (wristworn actigraph).

## Methods

### Participants

Adolescents between the ages of 11 years 0 months and 17 years 11 months were recruited through online and school advertisements, a participant contact database maintained by the Duke ADHD (Attention-Deficit/Hyperactivity Disorder) Program, and word of mouth. Inclusion criteria included the ability to follow written and verbal instructions in English, access to a smartphone in order to complete daily sleep diaries, and ability to comply with all study requirements and procedures. Because we were interested in evaluating feasibility of wearable sleep EEG device use among typical adolescents drawn from the community (rather than a clinical sample), participants were excluded if they indicated a diagnosis of sleep apnea or periodic leg movement syndrome, current use of prescribed or over-the-counter sleep aids (eg, sedatives, melatonin), diagnosis of an acute or chronic medical illness or use of medication that may interfere with sleep as determined by the research team, and/or inability to comply with study requirements. If subjects were currently taking melatonin supplements on an as-needed basis (ie, not daily), they were given the option to initiate a 7-day washout period in order to participate in the study (n=2). Participants and their guardians were compensated up to $200 for completing all study-related activities. Compensation was not based on the usability of data provided by participants. This study was approved by the Duke University School of Medicine institutional review board and registered with ClinicalTrials.gov [NCT03843762], and the study protocol was conducted according to Good Clinical Practice standards.

### Procedure

After passing a brief prescreen assessment via telephone, participants attended an in-person intake visit accompanied by a parent or legal guardian. During the intake visit, participants and guardians signed an electronic informed consent form. Verbal assent was obtained from children younger than age 12 years. Eligible participants were provided with a sleep EEG device and actigraph and given detailed instructions on how to use and care for both devices. Specifically, participants were instructed to wear the actigraph on their nondominant wrist 24 hours a day for the study duration unless the device was going to be submerged in water, in which case participants were asked to remove the actigraph for as little time as possible. In order to ensure that the sleep EEG devices were used properly, a study coordinator reviewed the step-by-step instructions located in the device manual with each participant. Participants were also provided with a copy of the manual and directions on how to locate additional instructional videos on the manufacturer’s website.

Sleep recordings were collected for 1 week beginning the evening of the in-person intake visit. In addition to using the wearable devices, participants also completed an electronic sleep diary each morning via Research Electronic Data Capture (REDCap), a secure web platform. The link to the daily sleep diary was sent each morning via text message, and an evening reminder was sent automatically if the diary was not completed. As is standard procedure for actigraphy [13], the sleep diary was used to ensure completeness of data collected by the wearable sleep devices. On day 8 of using the wearables, participants and their guardians completed an in-person follow-up visit, during which the actigraphs and sleep EEG devices were collected and adolescents completed a survey querying device acceptability via REDCap.

### Measures

#### Wearable Sleep Devices

Sleep EEG was collected using a Zmachine Insight+ device (General Sleep Corporation). The Zmachine Insight+ is a single-channel EEG device with accompanying software that provides sleep staging scoring [25,26]. Participants were instructed to place one disposable (ie, one-time use) sensor behind each ear and one on the center of their neck directly prior to getting into bed each evening, and remove the sensors immediately following their final awakening. Sensors were connected to the Zmachine Insight+ device via wires, and participants were instructed to place the device next to their bed on a bedside table or similar. Actigraphy was collected using a wGT3X-BT (ActiGraph LLC) worn on participants’ nondominant wrists. These actigraphic devices are easy to use, acceptable to adolescents, sufficiently reliable and valid to assess sleep behavior, and commonly used in clinical research settings [13]. The daily Consensus Sleep Diary queried previous night bedtime, sleep-onset latency, wake time during the night, time of final awakening, and final rising time [31].

#### Feasibility and Acceptability of Wearable Sleep Devices

Feasibility was evaluated by the rate of study completion and the number of nights with viable data (full/complete recordings). Acceptability was assessed using the Wearable Acceptability Survey for Sleep (WASS; Multimedia Appendix 1) developed by authors JRLA and ADK. This 10-item scale is designed to optimize face validity and measures comfort of wearable sleep devices while asleep and awake, sleep disturbances resulting from device use, and satisfaction with the devices. The first 3 questions were adapted from those developed by ADK and colleagues for a previous study [32], and the scale was further expanded upon with 7 additional items by JRLA and ADK [33]. Acceptability of sleep EEG and actigraphy were queried separately. Internal consistency of the scaled items (items 1, 2, 3, 7, and 8) was high when queried about actigraphy (Cronbach alpha=.79) and EEG (Cronbach alpha=.80). This measure has not been previously published.

## Results

### Demographics of the Sample

A total of 123 adolescents and their guardians expressed interest in study participation and completed an eligibility prescreening via telephone. Of these individuals, 3 were excluded due to use of sleep medications and 13 declined to participate in the study following the phone screen. A total of 107 participants completed the initial in-person visit. One individual attended the in-person visit but declined participation following the consent process. Two additional individuals completed the study in full but are not included in this analysis due to difficulty retrieving the devices attributable to circumstances surrounding the novel coronavirus (ie, North Carolina COVID-19 shelter-in-place order), leaving a final sample of 104. See Table 1 for a summary of the demographics of the sample, which is reflective of the population demographics of Raleigh-Durham-Chapel Hill, North Carolina.

**Table 1:** Demographics of the study sample (n=104).

Characteristic		Value	
Age in years, mean (SD)		14.43 (1.77)	
Sex, female, n (%)		49 (47.1)	
**Race, n (%)**			
	African American		29 (27.9)
	White		67 (64.4)
	Asian		3 (2.9)
	Native American		1 (1.0)
	More than 1 race		4 (3.8)
**Ethnicity, n (%)**			
	Hispanic		7 (6.7)
	Non-Hispanic		97 (93.3)

### Feasibility of Wearable Sleep Devices

All participants (100%) who enrolled in the study following the intake visit completed the study. Regarding the sleep EEG device, 94.2% (98/104) of adolescents successfully achieved at least 1 full night of sleep recording. Of these individuals, the mean number of nights of successful recording was 5.42 (SD 1.71), the median number was 6, and the modal number was 7. A total of 86.5% (90/104) successfully recorded at least 3 full nights of sleep EEG recording, the amount recommended for obtaining stable measures of sleep architecture physiology using EEG. Of the 6 adolescents who were unable to successfully record EEG data, 4 adolescents appeared not to comply with sensor app instructions, 1 adolescent decided not to wear the device due to initial discomfort, and 1 adolescent accidently broke the device while it was not in use. It is notable that among the adolescents who successfully recorded at least 1 full night of data but less than 7 full nights of data, sleep recording was typically attempted by the adolescent on the other nights but the recording was incomplete (there were partial recordings because, for example, wires became unplugged so recording stopped).

**Figure 1:**
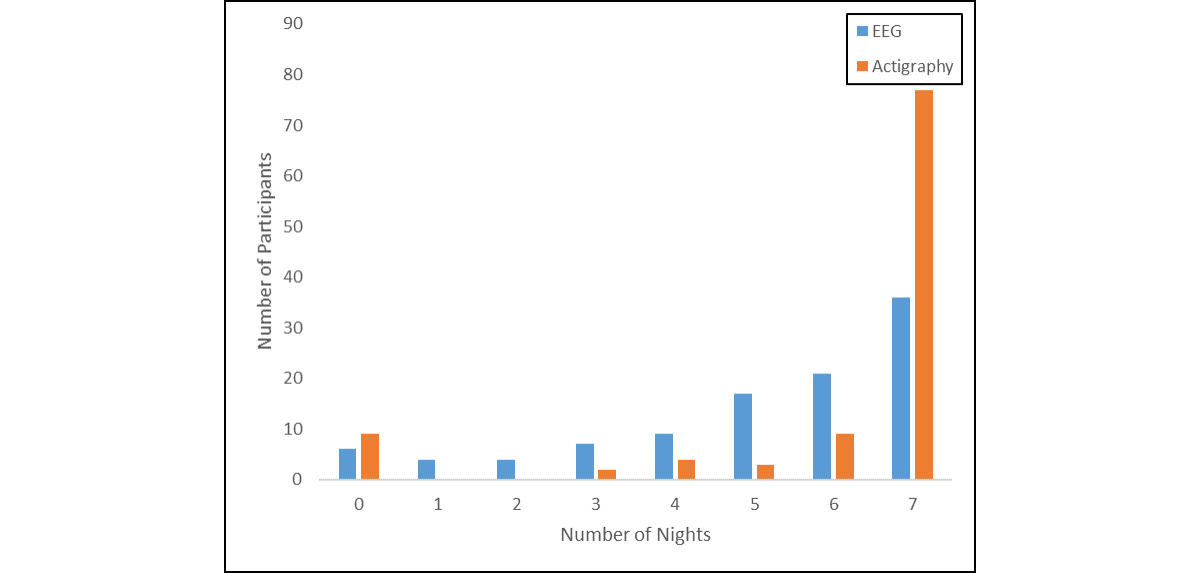
Number of nights of successful sleep recording by sleep device.

A similarly high rate of adolescents successfully recorded at least 1 full night of sleep recording using the wristworn actigraph device (95/104, 91.3%). Of these individuals, the mean number of nights of successful recording was 6.63 (SD 0.90), the median number was 7, and the modal number was 7. A total of 85.6% (89/104) recorded at least 5 nights of actigraphy sleep recording, the number of nights recommended for obtaining stable measures of sleep behavior using actigraphy. Of the adolescents who were unable to unsuccessfully record sleep data via actigraphy, the reasons were device/battery failure (5/9), lost device (2/9), and decision not to wear due to initial discomfort (2/9). See Figure 1.

### Acceptability of Wearable Sleep Devices

#### Comfort

**Figure 2:**
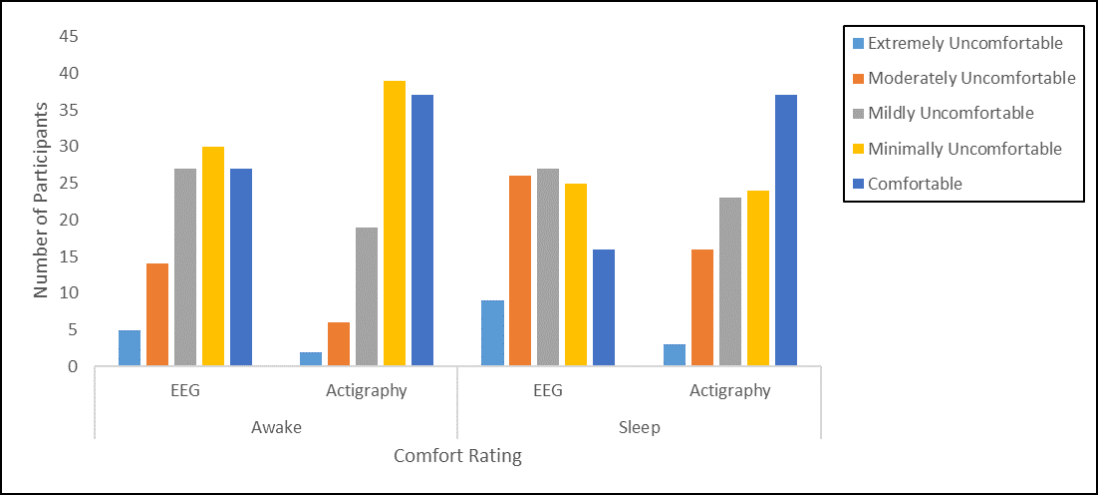
Ratings of comfort for sleep wearables while awake and sleeping.

The majority of adolescents rated both devices in the comfortable to mildly uncomfortable range while awake (sleep EEG: 81.6% (84/103); actigraphy: 92.2% (95/103) and sleeping (sleep EEG: 66.0% (68/103); actigraphy: 81.6% (84/103); Figure 2). When asked what led to discomfort when using the sleep EEG device, adolescents most frequently mentioned issues related to the wires (27/104; eg, getting caught/tangled or becoming detached/requiring reattachment due to movements during sleep) or the adhesive used to attach the sensors (28/104; eg, left residue or was itchy). A handful of participants mentioned discomfort due to difficulty changing sleep positions while wearing the device (5/104) and expressed concerns that they would mess up the recording while sleeping (3/104). When asked what led to discomfort while using the actigraph wristwatch, adolescents most often cited issues related to the light (18/104), which they found distracting while initiating sleep, and the wristband (26/104; eg, too loose or too tight, itchy/scratchy, too bulky). For both devices, it is notable that most adolescents rated any discomfort as minimal or mild.

#### Wearable-Related Sleep Disturbances

**Figure 3:**
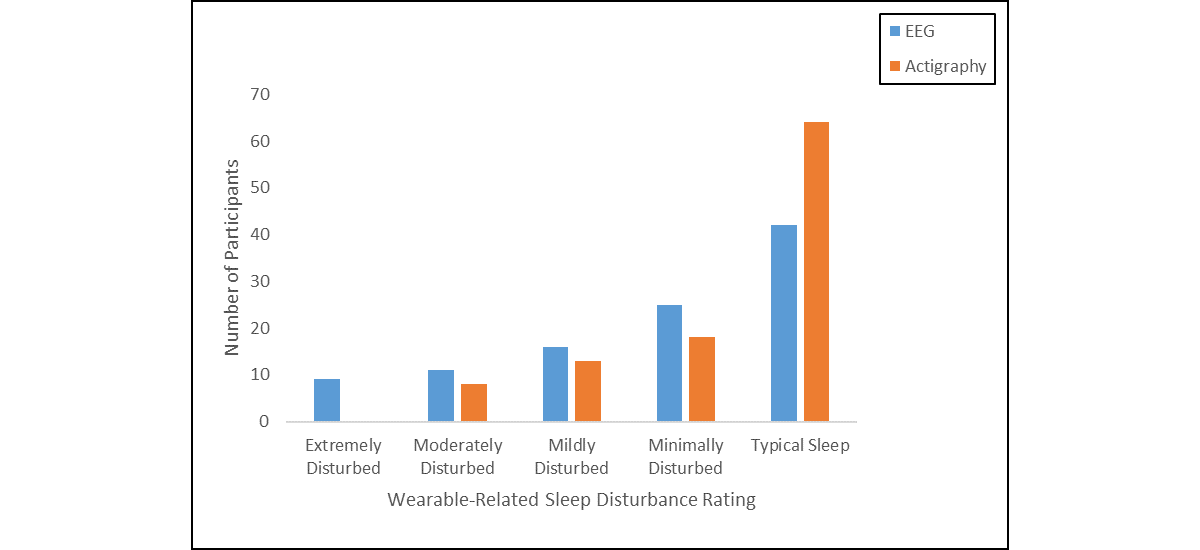
Wearable-related sleep disturbances by device.

Regarding perceived device-related interference with sleep, most adolescents indicated that their sleep was either typical of their normal sleep while wearing the sleep EEG device (42/103, 40.8%) or that the sleep EEG device resulted in minimal to mild sleep disturbances (41/103, 39.8%). Similarly, the majority of youth rated their sleep while wearing the wristworn actigraph as typical (64/103, 62.1%) or minimally to mildly disturbed (31/103, 30.1%). The most frequently reported device-related sleep disturbances due to EEG were difficulties falling asleep (24/104) and waking more frequently (23/104), followed by poorer sleep quality (18/104) and early waking (11/104). Most of the reported disturbances associated with the wristworn actigraph regarded difficulties falling asleep (20/104), followed by more night awakenings (7/104), poorer sleep quality (6/104), and early waking (6/104; Figure 3).

**Figure 4:**
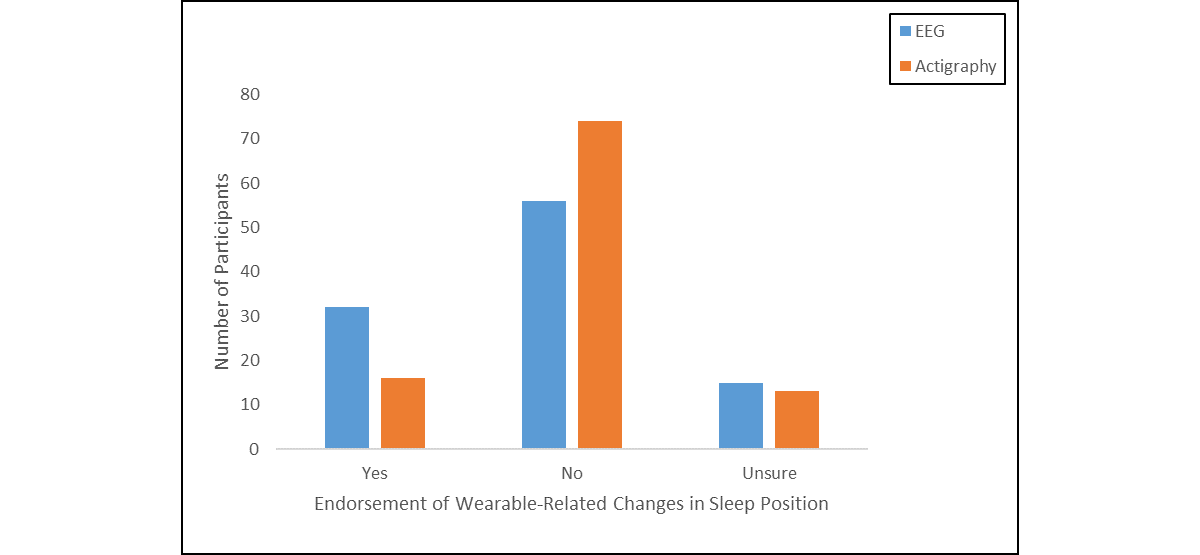
Endorsement of wearable-related changes in sleep position by device.

A minority of adolescents reported that they slept in a different position due to use of the sleep EEG device (32/104, 30.8%) or wristworn actigraph (16/104, 15.4%; Figure 4). Of the participants who endorsed a change in sleeping position due to sleep EEG, endorsements of preferred typical sleeping position included (participants could select more than one) sleeping on their back (3/32), stomach (5/32), side (13/32), changing positions frequently (18/32), or sleeping in all positions equally (4/32). Of the participants who endorsed a change in sleeping position due to actigraphy, endorsements of preferred typical sleeping position included sleeping on their stomach (3/16), side (6/16), changing positions frequently (9/16), or sleeping in all positions equally (1/16). These results suggests that across both devices, device-related changes in sleep position were most frequently endorsed by youth who already experience frequent position changes followed by those who sleep on their sides.

#### Satisfaction

**Figure 5:**
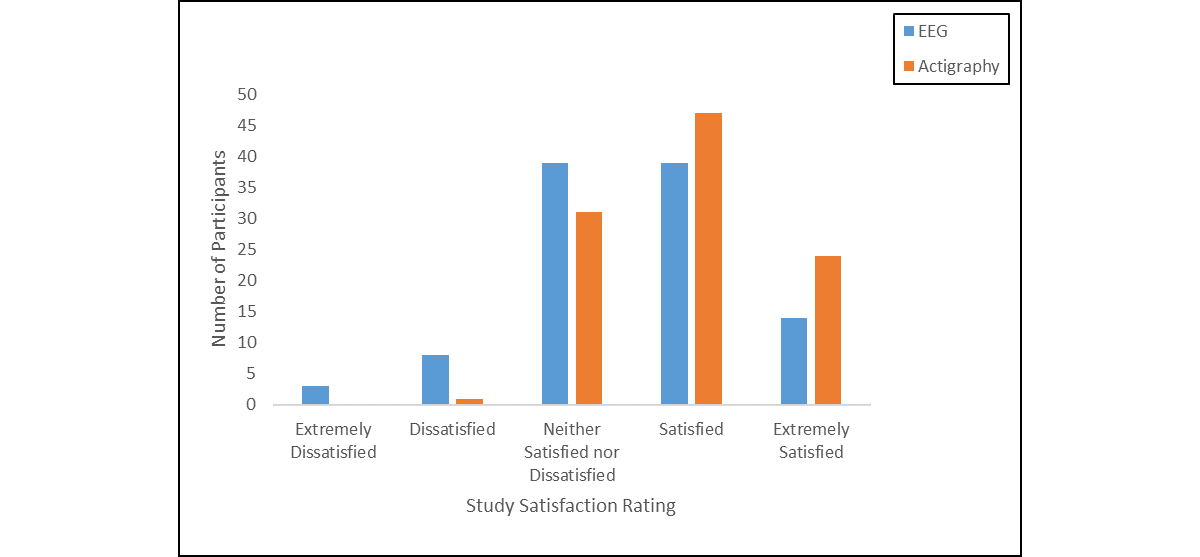
Overall study satisfaction by device.

Adolescents were most likely to rate themselves as satisfied with participation in the study based on their experience with the sleep EEG (53/103, 51.5%) and actigraphy (71/103, 68.9%) devices, followed by ambivalence (neither satisfied nor dissatisfied; 39/103, 37.9%, for the sleep EEG; 31/103, 30.1%, for the actigraph). Very few adolescents rated themselves as dissatisfied with their participation in the study based on experience with the sleep EEG (11/103, 10.7%) or actigraph (1/103, 1.0%; Figure 5).

Interestingly, there was a statistically significant association between satisfaction and device comfort while sleeping (χ^2^_4,n=103_=17.21, *P*=.002); the small number of adolescents who rated themselves as dissatisfied with the study based on sleep EEG use were also more likely to have experienced significant (moderate to severe) device-related discomfort (number observed = 9, number expected = 3.74). Similarly, there was a statistically significant association between satisfaction and significant (moderate to severe) device-related sleep disturbances (χ^2^_4,n=103_=21.28, *P*=.002), such that adolescents who rated themselves as dissatisfied with sleep EEG use were more likely to have experienced significant device-related sleep disturbances (number observed = 9, number expected = 2.14).

**Figure 6:**
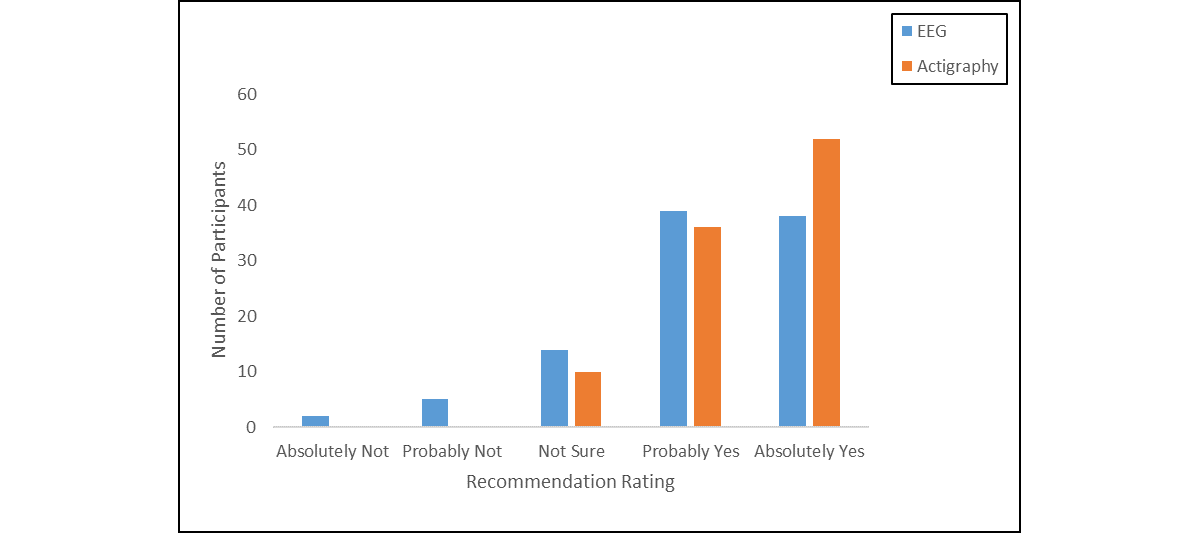
Number of adolescents recommending study to a friend by device.

The majority of adolescents would recommend the study to a friend based on their experience using the sleep EEG (77/98, 79%) and actigraphy (88/98, 90%; Figure 6).

## Discussion

### Principal Findings

Despite its importance in supporting overall health, sleep is infrequently evaluated during adolescence, a period characterized by shifts in sleep architecture and behavior. Adolescence has also been identified as a critical period for initiating lifelong health behaviors with the potential for a significant, long-term impact for the individual and societal/health care systems. Wearable sleep EEG devices have the potential to overcome obstacles to sleep assessments in routine health care settings and may allow for evaluation and treatment at a population level. However, to date, the usability of such devices in this age group has been unknown. Our findings suggest that the vast majority of adolescents were able to successfully record multiple nights of sleep data using the sleep EEG device. In addition, while adolescents described some discomfort and device-related sleep disturbances associated with wearing the sleep EEG device, most rated any discomfort as minimal to mild. Very few adolescents described dissatisfaction with their experience in the study. Notably, this pattern of results was similar to the pattern observed when using a commonly administered wearable (wristworn actigraph) assessing sleep behavior for this age group although satisfaction was numerically higher and discomfort and disturbances numerically lower for the actigraph compared with the sleep EEG device. In addition, it is notable that sleep EEG device-related disturbances were more likely to be endorsed by adolescents who sleep in certain sleep positions (eg, side sleepers, individuals who already change positions frequently), suggesting some individuals may be better suited to use the device than others.

These results have implications for the use of wearable sleep EEG devices with adolescents in both clinical and research settings. First, although wristworn activity monitors have utility for providing information about some aspects of sleep [12,13], EEG measures provide a more robust picture of sleep health by providing more accurate measures of sleep onset latency and night awakenings, particularly in those with sleep disturbance, and SWS and REM sleep, aspects of sleep architecture that have been associated with both psychiatric and physical disease in this age group [34]. For example, the suppression of SWS and REM sleep has been shown to be associated with insulin resistance in adolescents [35], less REM sleep has been found among adolescents with asthma [36], and early onset of REM and increased REM density may characterize the sleep of adolescents with depression [37]. Thus, sleep EEG measures have the potential to provide a more comprehensive representation of adolescent sleep as it relates to physical and mental health outcomes, and this study suggests that commercial grade wearables may provide a feasible, acceptable method for assessing sleep architecture in this age group. However, demonstration of feasibility and acceptability of sleep EEG among adolescents is only the first step toward incorporation of sleep EEG into routine clinical settings, as additional challenges (eg, validation of automated scoring algorithms, validation of findings from recording EEG data from forehead or mastoid versus standard placement of EEG electrodes within the hairline, training primary care physicians to dispense and interpret sleep EEG devices) would need to be addressed in order to facilitate meaningful clinical application.

Second, given costs associated with traditional PSG assessments, including not only the devices themselves but also access to a specialized sleep clinic and trained personnel, assessment of adolescent sleep architecture at a population level has been, to date, unattainable. Access to sleep EEG assessments in routine health care settings, including pediatric primary care, has been similarly limited. Wearable sleep EEG devices provide a potential opportunity to expand access to sleep evaluations for adolescents in both research and clinical settings, which may in turn allow for the acceleration of sleep-based mHealth apps in this age group. As mentioned above, interest in developing user-friendly, efficacious mHealth apps among adolescents is increasing at a rapid pace [8-10], in part due to adolescent engagement and facility with emerging technologies [38]. Incorporation of sleep EEG assessment via wearables may allow for refinement of mHealth apps targeting sleep in this age group—for example, by helping to identify adolescents most likely to benefit from sleep-based mHealth and/or to aid in monitoring sleep improvements resulting from such interventions.

### Future Directions

Results from this study offer many exciting new directions for future research. For example, although ambulatory PSG has been linked to health outcomes among adolescents, to date no studies have examined associations between consumer-grade sleep-EEG, which is scored using commercial algorithms and obtained from nonstandard scalp locations, and physical and psychiatric health in this age group. Given the importance of adolescence for shaping healthy behaviors across the lifespan, elucidating relationships between wearable sleep EEG and health outcomes is a critical next step for driving the development of mHealth apps targeting sleep and overall health in this age group. Second, future studies may examine the concordance between measures of sleep including consumer-grade sleep EEG, actigraphy, and self-report, as well as how these measures may be best used in conjunction to assess and support sleep health in this age group. Specifically, while sleep EEG has benefits in accurately assessing sleep stages, as a 24-hour measure of sleep behavior, actigraphy allows for the calculation of estimates of circadian patterns [39,40] as well as sensitive measures of the regularity of sleep patterns [41,42]. These findings suggest that both sleep EEG and wristworn wearables are feasible and tolerable in this age group and future studies should investigate how to optimize and integrate information collected from sleep wearables to enhance health outcomes among adolescents. Third, these findings may suggest avenues for improving sleep EEG technology. Namely, both feasibility and acceptability of sleep EEG devices in this age group were negatively impacted by specific features of the device, such as the wires, which contributed to both lost data (by becoming unplugged) and some, usually mild, discomfort. Wireless EEG systems have been developed and tested [43], and if successfully adapted, may have utility for assessing sleep in adolescents. Relatedly, unlike other commercial-grade devices, the device was attached to the head only via sensors rather than with a headband or cap [24]. The relative acceptability of these different features among adolescents is still an open question and may inform further refinement of sleep EEG systems. In addition, facilitating communication regarding sleep EEG data directly to adolescents and/or health care providers through smartphone apps or electronic health records may also support evaluation and treatments in this population and should be the focus of future work.

### Limitations

This study is subject to several limitations. First, this study focused on feasibility and acceptability of collecting sleep EEG data in generally healthy adolescents who might be seen in routine health care settings. Relatedly, the selected device did not measure respiratory function. Thus, the feasibility and acceptability of sleep EEG devices, including those with respiratory measures, in adolescent clinical populations (eg, obstructive sleep apnea), remain open questions. Second, it was beyond the scope of this study to assess the performance of wearable sleep EEG devices—for example, as compared with the gold standard sleep assessment (PSG). The selected device (Zmachine Insight+) has been shown to compare favorably with PSG in adults [26], and future studies should evaluate the performance of wearable sleep EEG devices in this age group.

### Conclusions

Wearable sleep EEG devices are feasible and acceptable for use with adolescents, a group characterized by developmental sleep changes and whose health behaviors may have a long-term impact on their well-being. Additionally, these devices may have utility in both clinical and research settings and for assessing and treating sleep disturbances at a population level. Future studies with adolescents should evaluate strategies for further improving usability of such devices, assess relationships between sleep EEG–derived metrics and health outcomes, and investigate methods for incorporating data from these devices into emerging mHealth interventions and apps.

## Appendix

Multimedia Appendix 1 Wearable acceptability survey for sleep.

## Abbreviations

ADHD: attention-deficit/hyperactivity disorder
EEG: electroencephalogram
mHealth: mobile health
PSG: polysomnography
REDCap: Research Electronic Data Capture
REM: rapid eye movement sleep
SWS: slow-wave sleep
WASS: Wearable Acceptability Survey for Sleep
