# Supplementary material for: Feasibility and Acceptability of Wearable Sleep Electroencephalogram Device Use in Adolescents: Observational Study
Source: JMIR Mhealth Uhealth. 2020 Oct 1;8(10):e20590. doi: 10.2196/20590 (PMC7563632; doi:10.2196/20590)
Supplement: Multimedia Appendix 1 [file mhealth_v8i10e20590_app1.docx]

## Appendix 1. Wearable Acceptability Survey for Sleep (WASS)

| **Rate the comfort of the device while AWAKE** (e.g., during the day)**:**   1. Extremely uncomfortable. 2. Moderately uncomfortable. 3. Mildly uncomfortable. 4. Minimally uncomfortable. 5. Comfortable. |
| --- |
| **Rate the comfort of the device while attempting to SLEEP:**   1. Extremely uncomfortable. 2. Moderately uncomfortable. 3. Mildly uncomfortable. 4. Minimally uncomfortable. 5. Comfortable. |
| **Rate how much the device disturbed your sleep:**   1. Extremely disturbed compared to my usual sleep. 2. Moderately disturbed compared to my usual sleep. 3. Mildly disturbed compared to my usual sleep. 4. Minimally disturbed compared to my usual sleep. 5. My sleep was typical of my usual sleep (no interference) |
| **If the device disturbed your sleep, please let us know how:** (Indicate **all** that apply)   1. I had more difficulty falling asleep than usual. 2. I woke up more frequently during the night than is typical. 3. I woke up earlier than usual and could not go back to sleep. 4. The quality of my sleep was poorer than usual (i.e., non-restorative sleep). 5. Other:_______________________________________________________ |
| **Indicate your typical preferred sleeping position:** (Circle **all** that apply)   1. Back 2. Stomach 3. Side 4. I switch positions frequently. 5. All positions equally comfortable. |
| **The device caused me to sleep in a different position than usual:**   1. True 2. False 3. Not sure |
| **Please rate your satisfaction with the sleep study based on your experience with the device.**   1. Extremely dissatisfied. 2. Dissatisfied. 3. Neither dissatisfied nor satisfied. 4. Satisfied. 5. Extremely Satisfied. |
| **Would you recommend the sleep study to a friend based on your experience with the device?**   1. Absolutely not. 2. Probably not. 3. Not sure. 4. Probably yes. 5. Absolutely yes. |
| **If you experienced discomfort as a result of the device, please let us know what led to the discomfort: _____________________________________________________________________________________** |
| **Is there anything else you would like us to know about your experience with the device? _____________________________________________________________________________________** |
